# Integrative oncology—the best from one world

**DOI:** 10.1007/s00432-022-04168-x

**Published:** 2022-07-01

**Authors:** Jutta Hübner

**Affiliations:** Klink Für Innere Medizin II, Universitätsklinikum Jena, Am Klinikum 1, 07747 Jena, Germany

Integrative Medicine is a terminus not well-defined. Most probably, when asking an integrative physician, you will get the answer that it is the combination of academic and complementary/alternative medicine—the best from two worlds.

This clearly is a misconception due to two reasons. First of all, it introduces two more ill-defined terms: complementary and alternative, which are often defined in a way that boundaries are floating as we will see later on. Second, a suggestive notion is hidden in the harmless word “combination”.

In this context, in the discourse on Integrative Medicine another word is used: pluralism. It refers to a combination of evidence-based diagnostic and therapeutic methods and of methods that are considered helpful by a group of experts, so-called empirical medicine. By this, most proponents of Integrative Medicine open the door for methods which are not based on evidence but on tradition, or as, for example, anthroposophist call it “singuläres Kausalerkennen” (singular recognition of causes). The contrast to evidence-based in this case often is named “cognition-based”. This is tempting for physicians as it restores the white coats—“I know what is good for the patient”. Moreover, groups of experts with the same “re-cognition” may unite in expert associations which for lay people including politicians, judges and journalists are hard to distinguish from science-based associations. Moreover, in case one points to the importance of evidence to put treatment decisions on a sound weighing of benefits and harms, one immediately will be pushed into the corner of the bad guys as one allegedly withheld patients natural methods.

The argument heard most often is that these “soft” methods (another not helpful term) at least don’t do harm. This most obviously is quite wrong as also soft methods (for example herbs or teas) may have side effects or interactions. Moreover, even in case of a placebo effect as with homeopathic globules, harm may be done by inducing false beliefs. These beliefs may in case of a cancer disease lead to a delay of treatment. Furthermore, these methods also do cost money either to the patient him/herself or to the society. The argument that homeopathy or acupuncture is less costly than many evidence-based treatments is not valid as in case of a serious disease the evidence-based treatment is still needed.

At first glance, there might be a place for placebo treatment for many ailments which are not life-threatening and normally underly self-healing as a common cold or headache. Many physicians and ethicists would consent. But is it really ethically justifiable that the trust of patients in the physician is deceived?

There is another argument, often cited for alternative medicine: the patients are asking for it. Yes, patients often ask for globules, consult acupuncturists or healers. However, when talking to those patients, you will never hear them telling that they purposefully choose methods with no more efficacy than placebo. No patient with cancer who takes globules does this with the notion to simply rely on self-healing.

Let us get back to the wording of complementary and alternative medicine—abbreviation CAM. Most often, they are defined like this.

Alternative medicine is any method which is used instead of a treatment recommended based on scientific evidence. In contrast, complementary medicine is used in addition.

Does this definition help? I do not believe so. If this were the case, one method may be alternative in one patient and complementary in another. Moreover, at least in oncology with multimodal treatment strategies: is “alternative” the case of a breast cancer patient who denies any treatment from operation to chemo- or radiotherapy to endocrine therapy? Or is it sufficient to be “alternative” that a patient after operation, chemo- and radiotherapy and 3 years of tamoxifen decides to use Cimicifuga instead of an aromatase inhibitor?

## I suggest another definition:

- Alternative medicine is any use of non-evidence-based method instead of or along with evidence-based treatment without even considering risks from side effects or interactions.
- In contrast, complementary medicine comprises additional, complementing methods which are evidence-based, are matched to the main evidence-based cancer treatment and for which the risk profile is well-known and considered in a benefit-risk assessment for the individual patient. Complementary medicine has different origins such as naturopathy, phytotherapy, mind–body-techniques. The aim of complementary medicine is to reduce side effects of cancer treatments, improve well-being and quality of life.

Therefore, what is the difference to supportive medicine and/or psycho-oncology? I am not sure, whether there is a strict distinction Yet, the unique quality of complementary medicine is that it is the answer to the patients’ question “What can I do by myself?”. In fact, this question is a strong offer from cancer patients to us physicians. And we might answer with an invitation: “I appreciate very much, that you are reflecting on this important issue.” And from this on, we might start communicating on what cancer is, how cancer develops and how modern treatments work. We might explain side effects and supportive treatments, we might involve a psycho-oncologist and we might start training our patients to be self-efficacious in using ginger tea against nausea (without forgetting to take anti-emetics) or choosing between honey, sage, peppermint or chamomilla against mucositis, doing a relaxation exercise or mindfulness practice or just have a walk with the dog or go playing with the grandchildren (which might also help against cognitive dysfunction and fatigue).

Therefore, in case you now think that all this is nice stuff in comprehensive cancer centres with a department on integrative oncology—no, simply keep in mind that the most natural methods we all have access to are eating and being physically active. Thankfully, these are the fields of complementary medicine with the best evidence and any physician or nurse should not only know this but should also talk on it with every patient.

Last and most important: what is Integrative Medicine? We just have described it: take the best from the world of evidence: evidence-based treatments, add evidence-based supportive medicine, evidence-based psycho-oncology, evidence-based nutrition and sports as well as evidence-based complementary medicine, communicate at your best and as often as possible with the patient and his next-ones. Remember that you are the human person that is the patient’s guide in a most unsettled time of his/her life. Last but not least, you are also the guide for those at the end of their patient journey who think about stopping treatment that provides more harm than benefit. In this case—and please start it in just time: palliative care improves survival and quality of life. Remember: the victory march of homeopathy was not due to any evidence of efficacy but to being less harmful than the traditional methods of bloodletting and others.

